# Application of a multicomponent model of convectional reaction-diffusion to description of glucose gradients in a neurovascular unit

**DOI:** 10.3389/fphys.2022.843473

**Published:** 2022-08-22

**Authors:** Yaroslav R. Nartsissov

**Affiliations:** Department of Mathematical Modeling and Statistical Analysis, Institute of Cytochemistry and Molecular Pharmacology, Moscow, Russia

**Keywords:** reaction-diffusion, neurovascular unit, blood flow, nutrients, blood–brain barrier

## Abstract

A supply of glucose to a nervous tissue is fulfilled by a cerebrovascular network, and further diffusion is known to occur at both an arteriolar and a microvascular level. Despite a direct relation, a blood flow dynamic and reaction-diffusion of metabolites are usually considered separately in the mathematical models. In the present study they are coupled in a multiphysical approach which allows to evaluate the effects of capillary blood flow changes on near-vessels nutrient concentration gradients evidently. Cerebral blood flow (CBF) was described by the non-steady-state Navier-Stokes equations for a non-Newtonian fluid whose constitutive law is given by the Carreau model. A three-level organization of blood–brain barrier (BBB) is modelled by the flux dysconnectivity functions including densities and kinetic properties of glucose transporters. The velocity of a fluid flow in brain extracellular space (ECS) was estimated using Darcy’s law. The equations of reaction-diffusion with convection based on a generated flow field for continues and porous media were used to describe spatial-time gradients of glucose in the capillary lumen and brain parenchyma of a neurovascular unit (NVU), respectively. Changes in CBF were directly simulated using smoothing step-like functions altering the difference of intracapillary pressure in time. The changes of CBF cover both the decrease (on 70%) and the increase (on 50%) in a capillary flow velocity. Analyzing the dynamics of glucose gradients, it was shown that a rapid decrease of a capillary blood flow yields an enhanced level of glucose in a near-capillary nervous tissue if the contacts between astrocytes end-feet are not tight. Under the increased CBF velocities the amplitude of glucose concentration gradients is always enhanced. The introduced approach can be used for estimation of blood flow changes influence not only on glucose but also on other nutrients concentration gradients and for the modelling of distributions of their concentrations near blood vessels in other tissues as well.

## 1 Introduction

It is well-known that a brain is the most nutrient sensitive organ in a human body. The adult human brain is generally limited to the use of glucose to fuel biochemical processes. It has an extremely high demand in metabolites especially in glucose and oxygen (Dwyer, 2002; Jespersen and Østergaard, 2012). The adult human brain consumes 20% of the total energy in the body while it comprises only 2% of the body weight (Ashrafi and Ryan, 2017). Indeed, the delivery of the compounds is fulfilled by a vasculature network which provide intensive cerebral blood flow (CBF) (Nartsissov, 2017).

Any kind of CBF impairment is the cause of sever neurodegenerative disorders like dementia and ischemic stroke (Gursoy-Ozdemir et al., 2012). Certainly, the nutrient supply is directly forming by a convectional reaction-diffusion in brain parenchyma. However, despite an obvious relation, theoretical modelling of a blood flow dynamic and metabolites reaction-diffusion are usually accomplished separately. Moreover, the type of the considered processes is often simplified advisedly. For example, under essential symmetrical properties of the system the general three-dimensional model can be reduced into a one-dimensional radial model by averaging over the vertical and angular variables in cylindrical coordinates and derive the one-dimensional reduced model similar to the lumped model (Calvetti et al., 2015). Sometimes such a way of modelling can be applied for analysis (Aubert and Costalat, 2005). Despite a complexity of the considering tissue, a remarkable success has been recently achieved in modeling of drug delivery systems for treatment of cancer. Numerical modeling of convectional diffusion yields a magnetically controlled intraperitoneal drug targeting system as a solution to improve the drug penetration into the tumor (Rezaeian et al., 2022). Moreover, the simulation results suggest that the thermosensitive liposomal doxorubicin delivery system in smaller tumors is far advantageous than larger ones (Rezaeian et al., 2019). Furthermore, it was shown that a multi-scale computational model in evaluating nano-sized drug-delivery systems can be used as a step forward towards optimization of patient-specific nanomedicine plans (Kashkooli et al., 2022). These findings clearly indicate an ability of combined multyphysical modeling to be a useful tool for pre-clinical and biomedical investigations.

Nevertheless, to get a right conclusion about the regulation properties of the system and explain or predict different effects, one needs to use the experimental or model approaches including an appropriate design of the complex phenomena. In the present study a theoretical approach to description of a spatial nutrient concentration distribution near a blood vessel has been established. It is based on a combination of a direct CBF modelling in a blood capillary with a convectional reaction-diffusion of the metabolite in a surrounding brain tissue. An essential feature of the introduced approach is explicit consideration of the metabolite transport systems in endothelia cells, and astrocyte end-feet. Moreover, the represented design of the model makes it possible to distinguish the lumen- and tissue- orientated surfaces of the endothelial cells.

The represented design of the model adjusts to description of spatial-time gradients of glucose. The same scheme of a physical coupling with the transport systems on the surfaces may be implemented for other metabolites, like lactate, when the membrane transporters can provide a double-direction trans-membrane flow. For nutrients which have no especial transport systems, the approach also pertains, but the main difference will be in the absence of flux dysconnectivity functions on the internal boundaries. The examples of such nutrients are oxygen, nitric oxide, and some xenobiotic drugs. The advantage of the created approach with respect to estimation of the non-steady state metabolites gradients is the fundamental modification of boundary conditions. Indeed, when reaction-diffusion is modeled near a blood vessel the simplest way is to fix the concentration or fluxes on the boundaries corresponding to endothelium layer. However, in such a case the gradients will be unsensitive to the alterations of CBF. The introduced method helps to resolve this problem. Both hemodynamic and a convectional reaction-diffusion are explicitly coupled in a single project model. The introduced approach may examine alterations of metabolite concentrations gradients caused by time-scaled changes of CBF. The developed model is considered by the example of a neurovascular unit (NVU) with respect to diffusion of glucose.

## 2 A structural organization of neurovascular unit

Since turn of this century, it has been becoming clear that neurons, glia and microvessels are organized into well-structured anatomical formations which are involved in the regulation of CBF (Abbott et al., 2006). The brain is sheltered from the changing metabolite concentrations in blood by the obstacle which is called a blood–brain barrier (BBB). It surrounds the central nervous system (CNS) including the spinal cord (Hawkins et al., 2006). A selective ‘physical barrier’ is formed by the complex tight junctions between adjacent endothelial cells. They force most molecular traffic to take a transcellular route across blood/brain contact, rather than moving paracellularly through the junctions, as in most endothelia (Abbott et al., 2006). Moreover, pericytes, astrocytic end-feet and extracellular matrix (ECM) components are also included into the BBB as the structural components (Keaney and Campbell, 2015). These barriers will of course present challenges for delivery of nutrients, essential for normal brain growth, metabolism and function (Hladky and Barrand, 2018). While endothelial cells form the vessel walls, pericytes are embedded in the vascular basement membrane and astrocytic processes almost completely ensheath brain capillaries (Abbott et al., 2010). The endothelial layer is surrounded by a basement membrane and pericytes all closely enveloped by astrocyte (glial) end-feet (Hladky and Barrand, 2016). The pericytes have a contractile function as well as a role in inducing and maintaining barrier properties (Berthiaume et al., 2018). There are also nerve cells close by within the parenchyma. Finally, this whole assembly is called the neurovascular unit (NVU) (Hladky and Barrand, 2018). The role of glial cells is very essential, and it should be even reasonable to use a term “neuro-glial-vascular unit.” However, during our further explanation a classic term NVU will be used because the sense of coupling in the model is to combine a convectional reaction-diffusion of metabolite (neuro including both neurons and glia cells) and hemodynamics (vascular).

**FIGURE 1:**
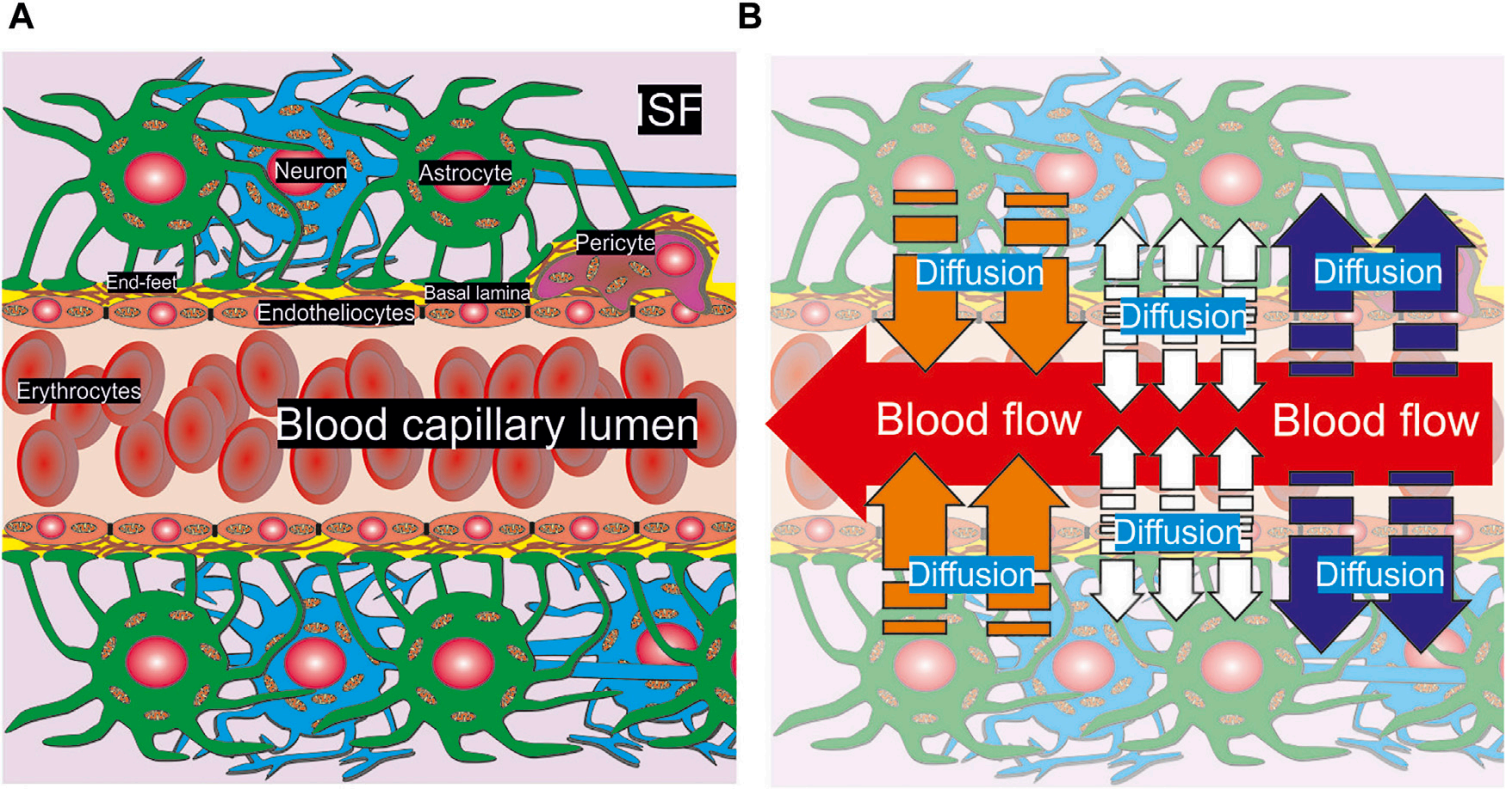
A scheme of anatomical composition of NVU. A part of a capillary pipe with erythrocytes included inside is surrounded by basal lamina, a pericyte, astrocytes and neurons **(A)**. The cells are pictured with the nucleus and mitochondria. End-feet are represented as the touch area near basal lamina and the pericyte. The physical processes of convection and diffusion are shown in appropriate areas of NVU **(B)**. A convectional CBF is represented as a red arrow in the capillary lumen. The diffusion of nutrients out/into the blood stream is indicated by blue and orange arrows respectively. The diffusion from the endothelium is marked by white arrows.

At the present stage of a detailed geometry, there is no difference between glia cells and neurons in the medium of convectional reaction-diffusion of metabolites. The parameters are considered as the average values of consumption and diffusion. One should remark that the same structure appears under any level of vasculature bifurcation in brain but for large and medium arteries a smooth muscle wall must be considered as an external cover of endothelial cells. However, due to a relatively high CBF rate and the multilevel boundary structure there is no real diffusion of glucose and other nutrients form the vessels with diameter more than approximately 60 μm. Thus, for description of CBF and metabolites convectional reaction-diffusion coupling a structure of capillary type NVU can be properly used. A summarized scheme of NVU is represented in Figure 1A.

It is very essential that the processes in a brain parenchyma near a capillary are clearly structured. On the one hand, blood is coming inside a capillary lumen supplying different chemical compounds. This process is described in terms of the fluid flow dynamics (Pontrelli, 1998) and it is a subject of experimental and theoretical studying of microvascular perfusion (Davis et al., 2008; Jensen and Chernyavsky, 2019). On the other hand, the nutrients can penetrate out or into a blood stream. For small arterioles, pre-capillaries, and capillaries the diffusion will be directed out of lumen (blue arrows, Figure 1B). On the contrary, for veins the opposite direction of metabolites transport dominates (orange arrows, Figure 1B). Some chemical compounds can be synthesized in endothelium cells and then they will spread out in both directions into capillary lumen and brain parenchyma (white arrows, Figure 1B). The example of such a compound is nitric oxide. The introduced approach yields evaluation of nutrients gradients for all possible considered conditions, because CBF and convectional reaction-diffusion are considered explicitly. Nevertheless, the calculations are fulfilled for the example of glucose diffusion.

## 3 A mathematical description of convectional reaction-diffusion in neurovascular unit

According to consideration of a complex biological structure mentioned in the previous Section A combined multiphysics approach needs to be used for description of the spatial non-steady state gradients of glucose in NVU. For successful modeling one has to consider different processes which provide evident influence on a glucose level in brain parenchyma. There is a successful example where a combined model has been applied to description of a novel drug delivery system based on the use of acoustic waves and temperature-sensitive liposomes. Herein, using an acoustics-thermal-fluid-mass transport coupling model, it was shown that the effective drug penetration into the tissue increased by 56% compared to conventional drug delivery (Sedaghatkish et al., 2020). In the present study, a mathematical model which describes physical phenomena near the inside/outside space of a blood vessel will be a combination of the fluids flow dynamics, diffusion, and kinetic consumption/production. In fact, the last ones are merged into reaction-diffusion and under a wide range of conditions the first process is included into the governing equation as an appropriate term.

Moreover, one should note that convection is also present in brain parenchyma. Despite a relatively low velocity as compared to CBF, it must be also regarded. Furthermore, a physiologically important role for local parenchymal convective flow in solute transport through brain extracellular space (ECS) is matched against diffusion even though it is not finally approved (Jin et al., 2016). Thus, the model of NVU is proposed as combined consideration of a blood flow and convectional reaction-diffusion for a metabolite both inside the capillary and in a surrounding tissue.

### 3.1 The geometry of the considered area of neurovascular unit

**FIGURE 2:**
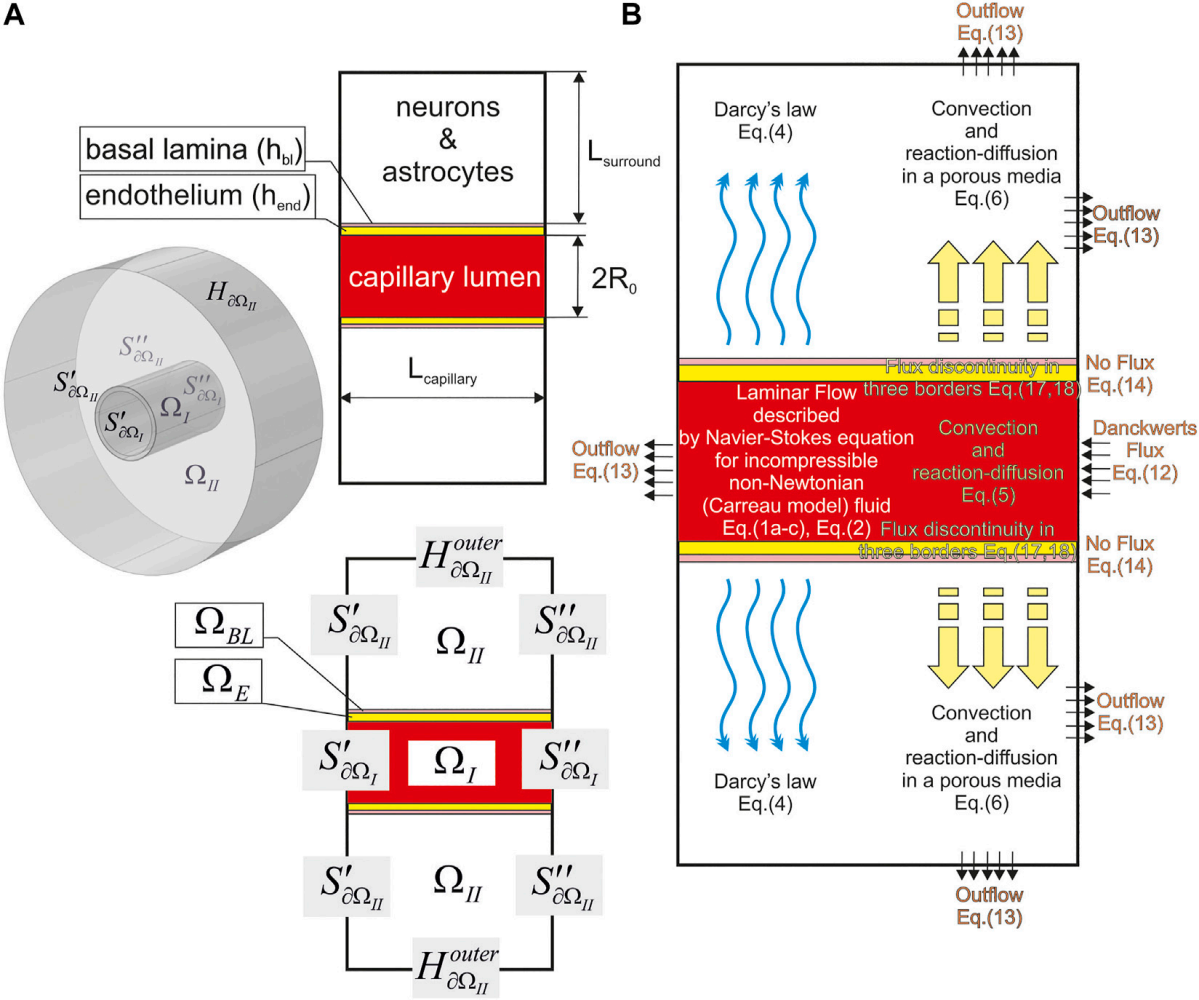
A geometry of the used digital NVU phantom. The main parameters are indicated on the plane projection **(A)** and the indication of the phantom parts and surfaces are shown both in an isometric drawing [The left part of **(A)**] and the plain view [The bottom part of **(A)**]. It is assumed that $H_{\partial \Omega_{I}}=H_{\partial \Omega_{E}}^{inner};H_{\partial \Omega_{E}}^{outer}=H_{\partial \Omega_{BL}}^{inner};H_{\partial \Omega_{BL}}^{outer}=H_{\partial \Omega_{II}}^{inner}$ . The distribution of physical processes with referenced number of the equations is represented in all parts of Ω **(B)** (see the text).

Initially, the geometrical shape of the modelled area must be determined. For this purpose, one needs to create a virtual (digital) phantom ( Ω ) which is in fact represent a combination of digital areas corresponding to biological prototype. A term “phantom” is used here as a description of the object where further numerical calculations will be fulfilled. It has a direct similarity with a physical imitation of the object during ionizing irradiation research. According to biological features of NVU described above (see Section 2.), all structures have been placed one after another (Figure 2A). A capillary lumen is represented as a short tube ( $\Omega_{I}$ ) with a circular section and the length of L_capillary_. Endothelial cells line the vessel intima, and they are regarded as a cylinder ( $\Omega_{E}$ ) with the fixed thickness of h_end_. The next cover is basal lamina which is also formalized as a thin cylinder ( $\Omega_{BL}$ ) with the width of h_bl_. For simplification, the line segment of a capillary without pericytes is used for modelling.

The rest part of NVU is approximated as a cylinder ( $\Omega_{II}$ ) surrounding all structures described above. This cylinder is supposed to consist of different cells. Astrocytes inhere in the first layer forming the end-feet contacting with basal lamina. In the further space neurons and astrocytes compose a heterogenic structure containing cells and interstitial fluid organized in sheets and tunnels. It was anatomical proved that diffusion distances to neurons and glial cell bodies for solutes and drugs are short because no brain cell is further than about 25 μm from a capillary (Abbott et al., 2010; Gould et al., 2017). Based on the indicated observation, the thickness of tissue-cylinder $\Omega_{II}$ is fixed on this value (L_surround_). For each type of the considered area the whole surface will be indicated as ∂Ω and the transverse and longitudinal surfaces as $S_{\partial \Omega }$ and $H_{\partial \Omega }$ respectively. The distances between neighbor end-feet are modelled by a division of $H_{\partial \Omega_{II}}^{inner}$ on two parts. One of them corresponds to the astrocytes processes with area $S_{end-feet}$ . Another one represents a cleft-area where a free diffusion occurs. Due to the system has a cylindrical symmetry the division of $H_{\partial \Omega_{II}}^{inner}$ should be made by intermittent circular stripes. The set of gemetrical parameters of the virtual phantom is represented in Table 1.

**TABLE 1:** The geometrical parameters of a NVU virtual phantom used for the modelling.

Symbol	Parameter	Value	Source
L_capillary_	The length of the considered capillary part	25 μm	According to the experimental limitations (Abbott et al., 2010)
R_0_	A capillary lumen radius	7 μm	Based on a possible upper range (Fleischer et al., 2020)
h_end_	The fixed thickness of the endothelial cells layer	1 μm	(Payne, 2004; Yazdani et al., 2019)
h_bl_	The thickness of a basal lamina layer	100 nm	(Arifin et al., 2009; Krüger-Genge et al., 2019)
L_surround_	A radial distance from $H_{\partial \Omega_{II}}^{inner}$ to $H_{\partial \Omega_{II}}^{outer}$	25 μm	According to the experimental limitations (Gould et al., 2017)

### 3.2 Modeling of the capillary blood flow

For many cases, the governing equation for the fluid flow is the non-steady-state Navier-Stokes equation for an incompressible flow without buoyancy effects (Huang et al., 2013; Iasiello et al., 2016)

$$\rho \frac{\partial \vec{u}}{\partial t}+\rho \left(\vec{u}\cdot \nabla \right)\vec{u}=-\nabla p+\nabla \left(\mu \left(\left|\dot{\gamma }\right|\right)\left(\nabla \vec{u}+{\left(\nabla \vec{u}\right)}^{Τ}\right)\right);$$ (1a)

$$\dot{\gamma }=2\varepsilon =\nabla \vec{u}+{\left(\nabla \vec{u}\right)}^{Τ},\left|\dot{\gamma }\right|=\sqrt{2\left(\varepsilon :\varepsilon \right)};$$ (1b)

$$\nabla \vec{u}=0;$$ (1c)

where $\vec{u}$ is the velocity vector along the coordinate system; ε denotes strain rate tensor; p, t and ρ are pressure, time, and the fluid density respectively. There is no slip on the boundary of the capillary lumen. The specific feature of this Eq. 1a for the non-Newtonian fluid flow is the dependence of the dynamics viscosity μ on shear rate $\left|\dot{\gamma }\right|$ .

There are a lot of time-dependent models proposed to describe thixotropic and viscoelastic behavior of blood (Yilmaz and Yaar Gundogdu, 2008). They designate the dependence of $\mu \left(\left|\dot{\gamma }\right|\right)$ directly. It was previously shown that the non-Newtonian nature of blood acts as a regulating factor to reduce the flow resistance and wall shear stress thereby considering shear thinning to have the most significant role in facilitating blood flow through stenotic vessels (Shukla et al., 1980). Moreover, blood is usually considered as a predominantly shear thinning fluid, especially under steady flow conditions (Fatahian et al., 2018). Thus, this property seems to have the most important non-Newtonian impact (Sochi, 2013). Having quickly considered a competitive analysis of the obtained modelled values in the literature, one is able to conclude that shear thinning is evaluated using the Carreau-Yasuda model, and yield stress is usually described by the Casson model (Yilmaz and Yaar Gundogdu, 2008; Sochi, 2013). As it was mentioned above shear thinning is the most important property in the description of hemorheology and hemodynamics.

Therefore, the Carreau model has been used for Eq. 1a in the present study. According to the Carreau model the dynamics viscosity is described by the following expression:

$$\mu \left(\left|\dot{\gamma }\right|\right)=\mu_{\infty }+\left(\mu_{0}-\mu_{\infty }\right){\left[1+{\left(\lambda \dot{\gamma }\right)}^{2}\right]}^{\left(n-1\right)/2}$$ (2)

where $\mu_{\infty }$ connotes a constant limit value when blood is treated as a Newtonian fluid, $\mu_{0}$ is the blood viscosity at a zero shear rate, λ is the time constant associated with the viscosity that changes with the shear rate, and n is an index parameter (Molla and Paul, 2012).

### 3.3 Numerical simulation of capillary blood flow changes

Although the exact value of p under a microcirculation level is controversial, the initial pressure in the capillary has been fixed at $p_{0}$ = 18.5 mmHg, and the difference of pressure Δp between inlet and outlet surfaces assigns CBF. The alteration in CBF is modelled by an application of a step-like function $f_{shift}^{dec/inc}\left(t\right)$ , which shifts both $p_{0}$ and Δp to the modified values. If one needs to consider a decrease of CBF, $f_{shift}^{dec}\left(t\right)$ will fall down to the fixed value which is lower than 0.5. For the example of an increased CBF the same function ( $f_{shift}^{inc}\left(t\right)$ ) raises up to the value 1.5.

$$\begin{array}{l} p_{inlet}=\left(p_{0}+\Delta p\right)f_{shift}^{dec/inc}\left(t\right);p_{outlet}=\left(p_{0}-\Delta p\right)f_{shift}^{dec/inc}\left(t\right);\\ f_{shift}^{dec}\left(t\right)=\left\{ \begin{array}{l} 1,t<t_{0};\\ 1-\delta \left(t\right),t\geq t_{0}; \end{array}\right.f_{shift}^{inc}\left(t\right)=\left\{ \begin{array}{l} 1,t<t_{0};\\ 1+\delta \left(t\right),t\geq t_{0}; \end{array}\right.\forall t:\delta \left(t\right)\in \left[0,a\right];0.5\leq a<1; \end{array}$$ (3)

**FIGURE 3:**
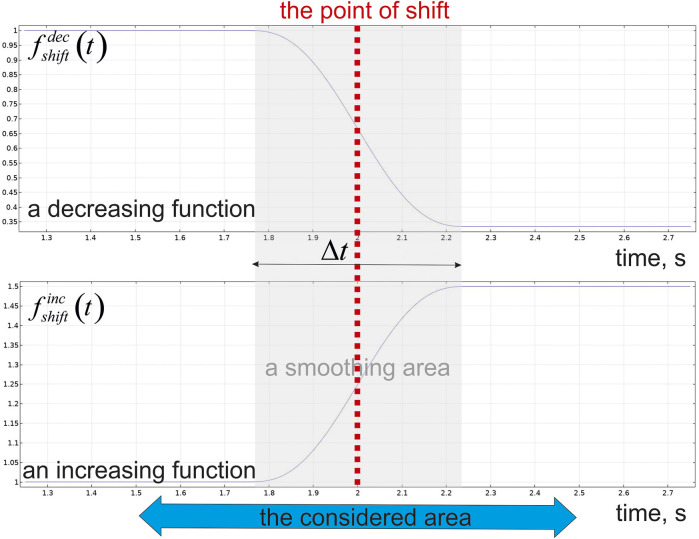
The step-like function governing inlet/outlet pressure in a capillary blood flow.

The view of $f_{shift}^{dec/inc}\left(t\right)$ is represented in Figure 3. The considered time is in a second’s range with the time-point of shift $t_{0}=2\;s$ . Indeed, the changes in a blood flow are always appeared with a continues lag phase, and a shift time-point is only a mid-point of the transition time area. This effect is modelled by a time dependent function ( δ(t) ) which helps to evaluate alteration of the pressure in the capillary fluently. The range of a smoothed area near the time-point of shift is characterized by Δt . It is taken as high as 50% of the considered area because the values are shifted smoothly from initial levels to final ones.

### 3.4 Modeling of a fluid flow in parenchymal extracellular space

Forming of metabolic gradients near a capillary surface obviously composes of diffusion and advection in the interstitial space which is facilitated by convection of interstitial fluid (ISF) in the paravascular space (Jin et al., 2016). To describe these processes the form of tissue structure geometry should be set explicitly. In some case it has been done on the base of 3D reconstructions of neuropil from electron microscopy images (Kinney, 2009; Mishchenko et al., 2010; Kinney et al., 2013). However, a digital form of the phantom is sometimes created based on the anatomical information. For example, to consider the glymphatic mechanism by modeling diffusive and convective transport in brain ECS, the geometry of the microvascular lobule can be idealized as a hexagonal lattice with a venule at the center, surrounded by six arterioles (Jin et al., 2016).

If the parenchymal structure is not assigned plainly the velocity of fluid flow in brain ECS can be estimated using Darcy’s law,

$$\vec{u}=-\frac{\kappa }{\mu_{ISF}}\nabla p$$ (4)

where κ and $\mu_{ISF}$ are permeability and dynamic viscosity of the interstitial fluid, respectively. The Eq. 4 is appropriate for description of convectional processes in $\Omega_{II}$ because it describes a flow in a porous media. This media includes conditions where ISF is incompressible fluid which is placed around the rigid bodies depicting neurons and glia cells. The set of the physical parameters used in the modelling of CBF and ECF is represented in Table 2.

**TABLE 2:** The main properties of theneeds to consider the diffusion of parenchyma medium and the characteristics of capillary blood flow. It should be stressed that there are a lot of measured values of ρ which are reported to blood. However, the most of them belong to the range from 1,029 kg/m^3^ (Gijsen et al., 1999; Kim et al., 2008), to 1,087 kg/m^3^ (Fan et al., 2009). Nevertheless, both a low (1,000 kg/m^3^), and a high density (1,410 kg/m^3^) are also described (Chen et al., 2006; Vimmr and Jonasova, 2008).

Symbol	Parameter	Value	Source
$\rho_{blood}$	Density of blood	1,070 kg/m^3^	Huang et al. (2013)
$\rho_{ISF}$	Density of an interstitial fluid	1,000 kg/m^3^	Zhan et al. (2017)
$\mu_{ISF}$	Viscosity of an interstitial fluid	7 × 10^–4^ kg/m/s	Zhan et al. (2017)
κ	Darcy’s permeability	6.5 × 10^–15^ m^2^	Arifin et al. (2009)
$\mu_{\infty }$	Limit viscosity of a Newtonian fluid	3.45 × 10^–3^ Pa × s	Molla and Paul. (2012)
λ	Time constant in the Carreau model	3.131 s	Molla and Paul. (2012)
$\mu_{0}$	Viscosity at a zero-shear rate	5.6 × 10^–2^ Pa × s	Molla and Paul. (2012)
n	Index constant in the Carreau model	0.3568	Molla and Paul. (2012)

### 3.5 Convectional reaction-diffusion of glucose in capillary and surrounding tissue

Having obtained the velocity field $\vec{u}$ using Eqs 1a, 4, one needs to consider the diffusion of glucose in the model. The equation governing the system describes several processes. They are diffusion, convection, and glucose consumption. The type of the equation depends on the considered area. For a blood stream, endothelium and the basal lamina large stain objects forming the obstacles are supposed to be insufficient. On the contrary, for parenchyma the most essential contribution is given by spatial heterogeneity of neurons and glia cells forming a typical porous media. Thus, the gradient of metabolites will be formed in the capillary lumen and two surrounding structures on the base of the following equation:

$$\frac{\partial c\left(\vec{r},t\right)}{\partial t}=\nabla \cdot \left(D\cdot \nabla c\left(\vec{r},t\right)\right)-\vec{u}\cdot \nabla c\left(\vec{r},t\right)+f_{con}\left(c\left(\vec{r},t\right),\vec{r}\right);$$ (5)

where c is the volume concentration of glucose, D is a diffusion tensor and $f_{con}\left(c\left(\vec{r},t\right),\vec{r}\right)$ is the function describing the rate of metabolic reactions which consume the diffusing molecules. There is no convection in $\Omega_{E,BL}$ . Thus, Eq. 5 will be applied in these areas with the zero second summand in the second member of equation. In the outside space diffusion is supplied by percolation of ISF into shears and tunnels of the parenchyma. It transforms Eq. 5 into the following form:

$$\frac{\partial }{\partial t}\left[\alpha_{ICF}c\left(\vec{r},t\right)\right]=\nabla \cdot \left(\left(D_{d}+\frac{\alpha_{ICF}}{\tau }D\right)\cdot \nabla c\left(\vec{r},t\right)\right)-\vec{u}\cdot \nabla c\left(\vec{r},t\right)+f_{con}\left(c\left(\vec{r},t\right),\vec{r}\right);$$ (6)

where $\alpha_{ICF}$ is porosity and it is also called volume fraction of ISF and τ is interstitial tortuosity. Additionally, the dispersion tensor $D_{d}$ for ISF has been included. It depends also on the fluid’s average velocity $\vec{u}$ in the void space,

$$D_{\left(d\right)ij}=\frac{1}{u}\sum_{k,l=1}^{3}a_{ijkl}u_{k}u_{l};u=\sqrt{\sum_{k=1}^{3}u_{k}^{2}};$$ (7)

where $a_{ijkl}$ is a property, called dispersivity, of the porous medium only, and $u_{k}$ is the k^th^ component of the fluid’s average velocity vector $\vec{u}$ (Bear, 1961). Since the early 60 s, almost all research on (solute) dispersion has been limited to isotropic porous media. For such media, the components of the dispersivity tensor have been shown to depend only on two material moduli, referred to as longitudinal ( $D_{\left(d\right)L}$ ) and transversal ( $D_{\left(d\right)T}$ ) dispersivities (Fel and Bear, 2010). In a common case the coefficients of transverse and longitudinal dispersion are non-linear functions of velocity:

$$D_{\left(d\right)L}=\frac{D_{c}}{\tau }+\alpha_{L}u^{n};\;\;\;D_{\left(d\right)T}=\frac{D_{c}}{\tau }+\alpha_{T}u^{n};\;\;\;n\in \left[1,2\right]$$ (8)

where the coefficients $\alpha_{L}$ and $\alpha_{T}$ are the longitudinal and transverse dispersivities, respectively, of the porous medium in the direction of transport, $D_{c}$ is a diffusion coefficient of substance c in the solvent and n is an empirically constant (Delgado, 2007).

Having assumed longitudinal dispersivity is proportional to a linear size of the system (L_surround_) (Pickens and Grisak, 1981) and taking into account the medium value of ISF in approximately 5 × 10^–7^ m/s (Zhan et al., 2017), one can neglect the second summand in a right part of Eq. 7. Thus, the dispersion transforms into:

$$D_{\left(d\right)L}=D_{\left(d\right)T}=\frac{D_{c}}{\tau };$$ (9)

Indeed, tensors in Eqs 5, 6 are the subject for experimental measurements. Despite the attempts to depict the true diffusion process, it was generally accepted that the gradients in a biological tissue be portrayed on a voxel scale of obtained images (Basser and Jones, 2002). In that case the physical diffusion coefficient has been replaced with a global, statistical parameter, the apparent diffusion coefficient (ADC) (Chanraud et al., 2010). The non-invasive observation of the water diffusion driven displacement distributions *in vivo* provides unique clues to the fine structural features and geometric organization of neural tissues, and the technique of brain tissue anisotropy measurement is actually based on a water diffusion magnetic resonance imaging (Le Bihan, 2003).

Taking together the mentioned above, the elements of diffusion tensors can be represented as production of the mean diffusion coefficient and anisotropy matrix.

$$D=D_{c}\cdot \left(\begin{array}{ccc} \sigma_{xx} & \sigma_{xy} & \sigma_{xz}\\ \sigma_{yx} & \sigma_{yy} & \sigma_{yz}\\ \sigma_{zx} & \sigma_{zy} & \sigma_{zz} \end{array}\right);$$ (10)

Certainly, in a common case it is difficult to indicate the sharp orientation of a short capillary part to parenchyma anisotropy. Nevertheless, without loss of generality one can suppose that $\sigma_{ij}=\delta_{ij}\cdot \xi_{ij}$ where $\delta_{ij}$ is Kronecker delta and $\xi_{ij}$ is a dimensionless value indicating the relative ratio between the directions. The physical parameters of diffusion in distinct parts of the considered virtual phantom ( Ω ) is represented in Table 3.

**TABLE 3:** The diffusion property of the medium. Diffusion coefficients for glucose considered as mean physical values which will be used as $D_{c}$ in Eq. 10 (Simpson et al., 2007).

Symbol	Parameter	Value	Source
$D_{Glc\;\;CBF}$	Glucose diffusion coefficient in CBF	3.1 × 10^–10^ m^2^/s	Kreft et al. (2013)
$D_{Glc\;BL}\;$	Glucose diffusion coefficient in basal lamina	1.6 × 10^–10^ m^2^/s	Bashkatov et al. (2003)
$D_{Glc}$	Glucose diffusion coefficient in astrocytes and neurons at 37°C	8.7 × 10^–10^ m^2^/s	Kreft et al. (2013)
$\sigma_{xx}$	Coefficient in diffusion/dispersion tensor	1	Le Bihan, (2003)
$\sigma_{yy}=\sigma_{zz}$	Coefficient in diffusion/dispersion tensor	0.33	Le Bihan, (2003)
$\alpha_{ISF}$	Porosity (volume fraction of ISF)	0.36	Vendel et al. (2019)
τ	Interstitial tortuosity	1.635	Sykova and Nicholson. (2008)

The third summand in the second member of Eqs 5, 6 is corresponds to consumption of glucose in the medium. As for many metabolites this process is mediated by an activity of enzymes transforming an initial substrate to the products of reaction. It should be noticed that the first step of the metabolic pathway for glucose oxidation is considered to be a unique reaction forming $f_{con}\left(c\left(\vec{r},t\right),\vec{r}\right)$ . Upon glucose has come to the cells, it is transformed into glucose-6-phosphate by hexokinase. It is generally considered that this first step of glycolysis has more flux control than its transmembrane transport. The activity of hexokinase is observed both in blood stream, endothelial cells and nervous parenchyma. Although the kinetic properties of such an enzyme is known well, the activity of the whole metabolic pathway should be taken into account. The rate equation of the hexokinase–phosphofructokinase system was proposed by Heinrich and Schuster (1996), but it should be extended by a term that accounts for the glucose effect according to classical Michaelis–Menten kinetics (Aubert and Costalat, 2005). The parameters

$$f_{con}\left(c\left(\vec{r},t\right),\vec{r}\right)=-\frac{\varepsilon_{Glc/ATP}^{\Omega_{I/E/II}}c\left(\vec{r},t\right)}{c\left(\vec{r},t\right)+K_{Glc}};\;\;\varepsilon_{Glc/ATP}^{\Omega_{I/E/II}}=\;k_{Glc}^{\Omega_{I/E/II}}c_{ATP}^{\Omega_{I/E/II}}{\left[1+{\left(\frac{c_{ATP}^{\Omega_{I/E/II}}}{K_{I,ATP}}\right)}^{nH}\right]}^{-1}\;\;\;\;\;\vec{r}\in \Omega_{I/E/II}\;\;$$ (11)

The Eq. 11 includes not only glucose concentration but also concentration of adenosine triphosphate (ATP) - $c_{ATP}^{\Omega_{I/E/II}}$ . The kinetic equation of steady-state hexokinase reaction depends on ATP concentration explicitly. However, due to the level of such a metabolite is essentially regulated in the living cells, it remains stable in a wide range of other parameters (Ainscow et al., 2002; Nartsissov and Mashkovtseva, 2006). Thereby, in the present study ATP concentration is considered as a constant and Eq. 11 is transformed to the classical simplified hyperbolic dependence. It is also supposed that endothelial cells and neuronal parenchyma have similar kinetic properties with respect to glucose consumption, but the level of hexokinase in blood cells is lower $\varepsilon_{Glc/ATP}^{\Omega_{I}}\ll \varepsilon_{Glc/ATP}^{\Omega_{E}}\approx \varepsilon_{Glc/ATP}^{\Omega_{II}}$ .

### 3.6 Boundary conditions and initial values

For any kind of partial differential equation problem, the boundary conditions are the most essential part which determines, in fact, quantitative characteristics of the solution. Moreover, the type of condition is related to the physical properties of the system. It is supposed that the capillary is essentially isolated from the external influence, and the main part of metabolites is coming with CBF. It means that on the end transverse surface of $\Omega_{I}$ Danckwerts condition has been set up:

$$\vec{n}\cdot \left(\vec{J}+\vec{u}\cdot c\left(\vec{r},t\right)\right)=\vec{n}\cdot \left(\vec{u}\cdot \tilde{c}\left(t\right)\right);\;\;\vec{r}\in {S^{\prime \prime }}_{\partial \Omega_{I}}\;$$ (12)

Another end transverse surface was validated with outflow condition:

$$\vec{n}\cdot \left(D\cdot \nabla c\left(\vec{r},t\right)\right)=0;\;\vec{r}\in {S^{\prime }}_{\partial \Omega_{I}}$$ (13)

Considering a convection field in ISF, the concentrations on $H_{\partial \Omega_{II}}^{outer}$ will be governed by Eq. 13 as well. Furthermore, appropriate end transverse surfaces of $\Omega_{E}$ and $\Omega_{BL}$ should be considered with an no flux condition:

$$-\vec{n}\cdot \vec{J}=0\;;\;\;\;\;\;\;\;\;\;\;\;\;\;\;\;\vec{r}\in {S^{\prime }}_{\partial \Omega_{E/BL}};\;\;\vec{r}\in {S^{\prime \prime }}_{\partial \Omega_{E/BL}}$$ (14)

If the virtual phantom length is long i.e., $L_{capillary}\gg L_{surround}$ and $c^{0}\left(t\right)∼\langle c\rangle$ then Eq. 14 can be replaced on the fixed boundary concentration.

$$c\left(\vec{r},t\right)=c^{0}\left(t\right);\;\;\vec{r}\in {S^{\prime }}_{\partial \Omega_{E/BL}};\;\;\vec{r}\in {S^{\prime \prime }}_{\partial \Omega_{E/BL}}\;$$ (15)

It should be stressed that a peculiarity of the brain tissue causes an evident effect of ISF on medium convection properties. Under considered conditions, a flow takes the metabolites away from the blood vessel boundary and it turns mathematical expression of Eq. 14 equal to Eq. 13 for $\vec{r}\in \left(\partial \Omega_{II}\backslash H_{\partial \Omega_{II}}^{inner}\right)$ . The difference between 13) and 14) will appear when the external flow of interstitial liquid is directed inside the virtual phantom. This case corresponds to veins type of a blood vessel, and it causes the substitution of Eq. 13 with Eq. 12 including an appropriate value of $\;\tilde{c}\left(t\right)$ .

The gradients of glucose will be always formed under circumstances of non-zero initial values of the metabolite. This essential feature of native compounds has a substantial difference with the same one to the toxic chemicals. Indeed, usual concentration of some mediator or a drug can be awfully close to zero because a high regulatory effect of the molecules needs to be appeared shortly. On the contrary, common participants in the metabolic pathways must be present in an essential amount in tissues all the time. It means that the initial concentrations of each metabolite are reasonably fixed at an average tissue level.

$$c\left(\vec{r},t\right){|}_{t=0}=\langle c\rangle {|}_{I/E/BL/II};\;\;\vec{r}\in \Omega_{I/E/BL/II}$$ (16)

In a common case the sharp values of initial concentrations are varied for different areas. However, the most diversity is observed between CBF and parenchyma.

### 3.7 Internal boundary discontinuities forming the flux dysconnectivity functions

The essential processes forming the gradients of metabolites near a capillary are the transport across the membranes. The transport of glucose is mediated by a well-known family of sodium-independent bi-directional facilitative transporters from the solute carrier 2 (SLC2) family of which 14 isoforms (GLUTs 1–14) are widely represented in endothelial cells, glia and neurons (Lizák et al., 2019). Despite variety of identified membrane carriers, GLUT1 and GLUT3 are the major glucose transporters in NVU (Patching, 2017). It is described by the usual equation of a passive transport. Under normal circumstances, as it was mentioned above brain glycolysis is not limited by glucose transport, but by phosphorylation of glucose to glucose-6-phosphate. Quantitative measurements suggest an asymmetric distribution of GLUT1 at the luminal and abluminal membranes and up to 40% of the GLUT1 protein may be sequestered within the cell cytoplasm at any given time (Patching, 2017). A number of other studies have quantified the relative amounts of GLUT1 in luminal and abluminal membranes and cytoplasm from humans and from other mammals with variable results (Deng and Yan, 2016).

The existence of the transmembrane transporters in endothelial cells and astrocytes end-feet forms an irregularity in a diffusion process. Indeed, having reached the surfaces of different areas, the molecules of glucose collide with an obstacle. There is no free diffusion available when they pass through $H_{\partial \Omega_{E}}^{inner}$ and $H_{\partial \Omega_{E}}^{outer}$ . For $H_{\partial \Omega_{II}}^{inner}$ there is a combination of the space with a free diffusion—the cleft between end-feet, and the membrane of astrocytes where the process of glucose entrance into the cell is the same as in endothelium. The phenomena described above form the internal flux irregularities in Ω which are introduced by the dysconnectivity functions.

These functions includes both kinetic properties of membrane transporters and their capacity on the surfaces $H_{\partial \Omega_{E}}^{inner}$ , $H_{\partial \Omega_{E}}^{outer}$ and $H_{\partial \Omega_{II}}^{inner}$ . It should be remarked that even though only one type of the transporter is considered but the formed fluxes are variated. Since the affinity of the protein to glucose is unchangeable under fixed conditions an obvious reason for such a diversity is the amount of GLUT incorporated into the membrane. A fluctuation of the typical value of GLUT content causes the quantitative multiplicity of the glucose transport in endothelium and end-feet. The combination of different GLUT yields the transmembrane flux of the metabolite. For each transporter, the rate equation is a typical hyperbolic function with maximal rate ( $V_{\max}^{k}$ ) and affinity ( $K_{m}^{k}$ ).

A superposition of the transport activity is defined as the following expression:

$$\begin{array}{l} -\vec{n}\cdot {\left(\vec{J}+\vec{u}\cdot c\left(\vec{r},t\right)\right)}_{H_{\partial \Omega_{E}}^{inner}/H_{\partial \Omega_{E}}^{outer}/H_{\partial \Omega_{II}}^{inner}}=\sum_{k}\frac{f_{GLUT\left(k\right)}\left(\vec{r}\right)\cdot V_{\max}^{k}\cdot c\left(\vec{r},t\right)}{K_{m}^{k}+c\left(\vec{r},t\right)};{|}_{\vec{r}\in S_{end-feet}}\;\;\;\\ -\vec{n}\cdot \left[{\left(\vec{J}+\vec{u}\cdot c\left(\vec{r},t\right)\right)}_{H_{\partial \Omega_{II}}^{inner}}-{\left(\vec{J}+\vec{u}\cdot c\left(\vec{r},t\right)\right)}_{H_{\partial \Omega_{BL}}^{outer}}\right]=0;\;\;\;\vec{r}\in H_{\partial \Omega_{II}}^{inner}\backslash S_{end-feet} \end{array}$$ (17)

The function $f_{GLUT\left(k\right)}\left(\vec{r}\right)$ in Eq. 17 describes a spatial distribution of GLUTs in the compartment’s membranes. It is validated in $H_{\partial \Omega_{E}}^{inner}$ , $H_{\partial \Omega_{E}}^{outer}$ and $H_{\partial \Omega_{II}}^{inner}$ . The first and the second surfaces contain the transporters in the endothelium cells and the last one comprises the GLUTs in end-feet of astrocytes. Generally, it is supposed that glucose transport in both barriers before parenchyma is mediated by GLUT1 isoenzymes (Weiler et al., 2017). According to this assumption the right part of Eq. 17 is transformed to

$$\sum_{k}\frac{f_{GLUT\left(k\right)}\left(\vec{r}\right)\cdot V_{\max}^{k}\cdot c\left(\vec{r},t\right)}{K_{m}^{k}+c\left(\vec{r},t\right)}=\frac{N_{GLUT1}^{H_{\Omega_{E}}^{inner}/H_{\Omega_{E}}^{outer}/H_{\Omega_{II}}^{inner}}\cdot k_{cat}^{1}\cdot c\left(\vec{r},t\right)}{N_{A}\cdot \left(K_{m}^{1}+c\left(\vec{r},t\right)\right)}$$ (18)

where $N_{GLUT1}^{H_{\Omega_{E}}^{inner}/H_{\Omega_{E}}^{outer}/H_{\Omega_{II}}^{inner}}$ is the number of carriers per μm^2^; $k_{cat}^{1}$ is the number of GLUT1 turnovers and $N_{A}$ is the Avogadro constant.

Finally, the kinetic constants and the parameters of transporters densities can be found in Table 4.

**TABLE 4:** The parameters of transmembrane glucose transport and the constants of consumption rates for glucose.

Symbol	Parameter	Value	Source
$K_{m}^{1}$	Glucose affinity constant of GLUT1	8 mmol/L	Simpson et al. (2007)
$N_{GLUT1}^{H_{\partial \Omega_{E}}^{inner}/H_{\partial \Omega_{E}}^{outer}}$	Number of GLUT1 in the endothelial membrane	1.0 × 10^3^ 1/μm^2^	Simpson et al. (2007)
$N_{GLUT1}^{H_{\partial \Omega_{II}}^{inner}}$	Number of GLUT1 in the end-feet membrane	0.018 × 10^3^ 1/μm^2^	Simpson et al. (2007)
$k_{cat}^{1}$	The number of GLUT1 turnover at 37°C	1.166 × 10^3^ 1/s	Lowe and Walmsley. (1986)
$k_{Glc}^{\Omega_{E/II}}$	Kinetic constant of hexokinase–phosphofructokinase system	120 × 10^–3^ 1/s	Aubert et al. (2001)
$K_{I,ATP}$	ATP inhibition constant on hexokinase–phosphofructokinase system	1 mM	Aubert et al. (2001)
$K_{Glc}$	Affinity constant to glucose	0.05 mM	Aubert and Costalat. (2005)
nH	Degree parameter of hexokinase–phosphofructokinase system	4	Aubert and Costalat. (2005)
$c_{ATP}^{\Omega_{I/E/II}}$	Concentration of ATP in distinct parts of the virtual phantom	1 mM	Köhler et al. (2020)

### 3.8 Computation and model evaluation

The model is created on the base of COMSOL Multiphysics ver. 5.5. All physical processes described above are included into structural geometry of NVU in appropriate way (Figure 2B). As it was mentioned above, the size of the blood vessel is taken as R_0_ = 7 μm. The set of concentration values is the following $\;\tilde{c}\left(t\right)=5\;mM$ , $\langle c\rangle {|}_{E/BL/II}=1\;mM$ , $\langle c\rangle {|}_{I}=5\;mM$ . The calculations have been evaluated using Intel^®^ Core ™ i9-7960X CPU 4.40 GHz and AMD Ryzen Threadripper 3990 × 64-Core Processor 4.3 GHz. The velocities fields and concentration gradients were obtained using the finite element method (FEM) applying User-Controlled Extra-Fine Meshes with minimum angle between boundaries in 240° and elemental size scaling factor 0.1. The governing equations of ISF (Eq. 4) and CBF (Eq. 1a) are solved to generate a flow field, and the velocities of both ISF and CBF are implemented into the reaction-diffusion equation with convection for transient simulations of the glucose gradients in blood flow (Eq. 5) and surrounding nervous tissue (Eq. 6), respectively. A mean calculation time was approximately 2.25 h for glucose estimations. The changes of CBF were simulated by a step-like function with a jump of pressures at t_0_ = 2 s (Figure 3).

The calculations have been made for the time range from t = 0 to t = 4 s. The considered area in a time scale is chosen as 1.5 s≤t≤2.5 s because the calculated values of blood flow velocities and glucose concentrations are changed in the smoothing area 1.75 s≤t≤2.25 s only. They eighter decrease or increase the base value $p_{0}$ and Δp with smoothing size of transitional zone in Δt = 0.5 s. The structure of NVU will be characterized by the ratio which describes the density of end-feet covering basal lamina. The parameter is calculated on the base of $H_{\partial \Omega_{II}}^{inner}$ division and it can be written as the following expression:

$$\delta_{end-feet}=\frac{S_{end-feet}}{S_{total}}\cdot 100\%.$$ (19)

The results of the modelling are both the velocity field in $\Omega_{I,II}$ and spatial-time distribution of the glucose concentration in all parts of the virtual phantom. If one needs to compare the results of modelling to the experimental data, the values are represented as a mean ± SD.

## 4 Results

**FIGURE 4:**
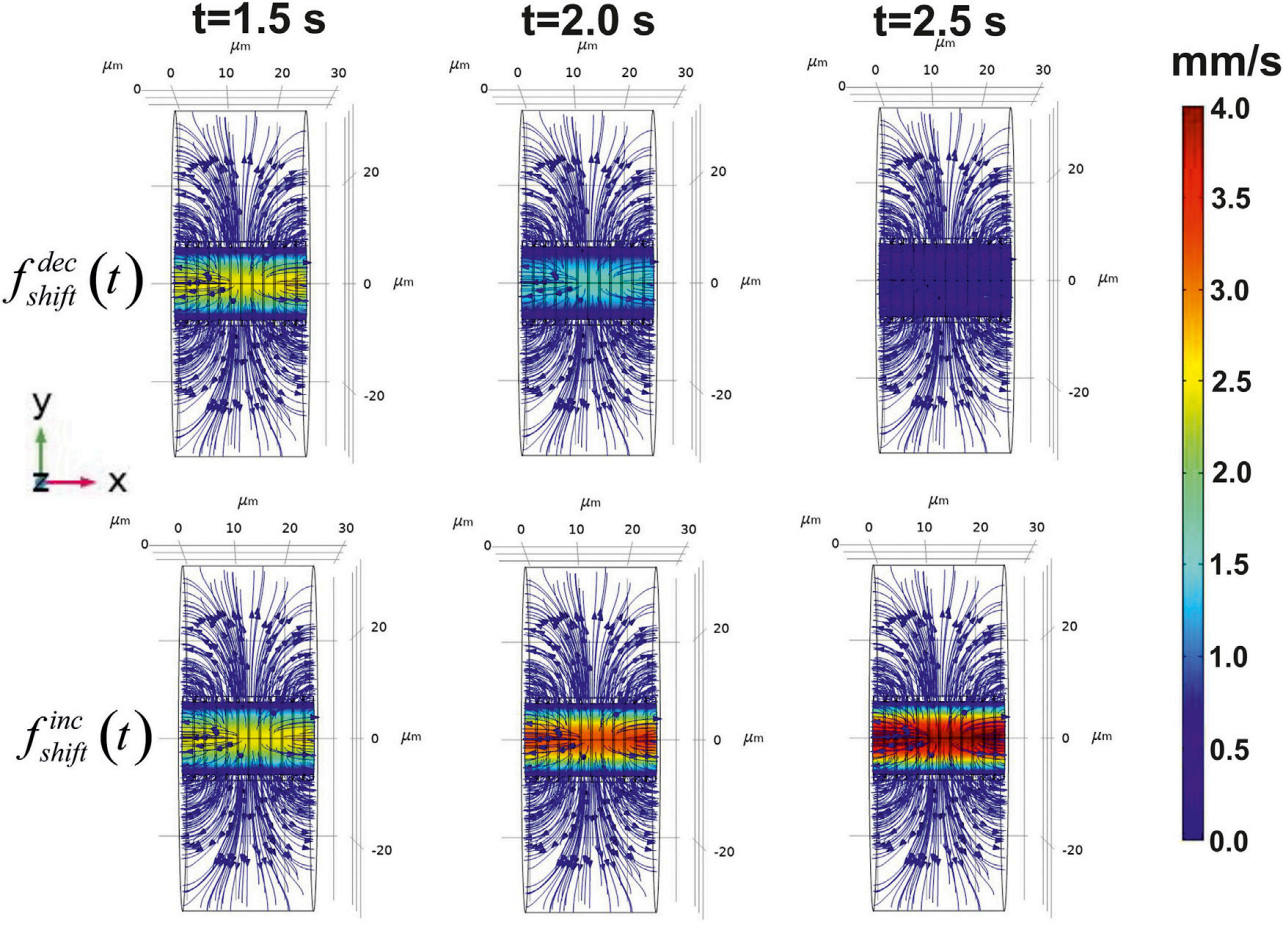
Calculated velocity field in the virtual phantom. The blood flow velocities and ISF flow are represented in the three points of the considered time area. The distribution of the velocities is shown on the central longitudinal cut plain XY. The application of the step-like function to the pressure causes either decrease of CBF ( $f_{shift}^{dec}\left(t\right)$ , upper set) or increase of CBF ( $f_{shift}^{inc}\left(t\right)$ , lower set). The arrows indicate the direction of calculated velocity fields in ISF.

The introduced approach makes it possible to evaluate both a velocity field in a blood stream/ISF convection and reaction diffusion of glucose in distinct parts of the considered phantom. Initially, the velocity field for CBF and convection in a nervous parenchyma part was obtained. The diagrams of this field are represented in Figure 4.

**FIGURE 5:**
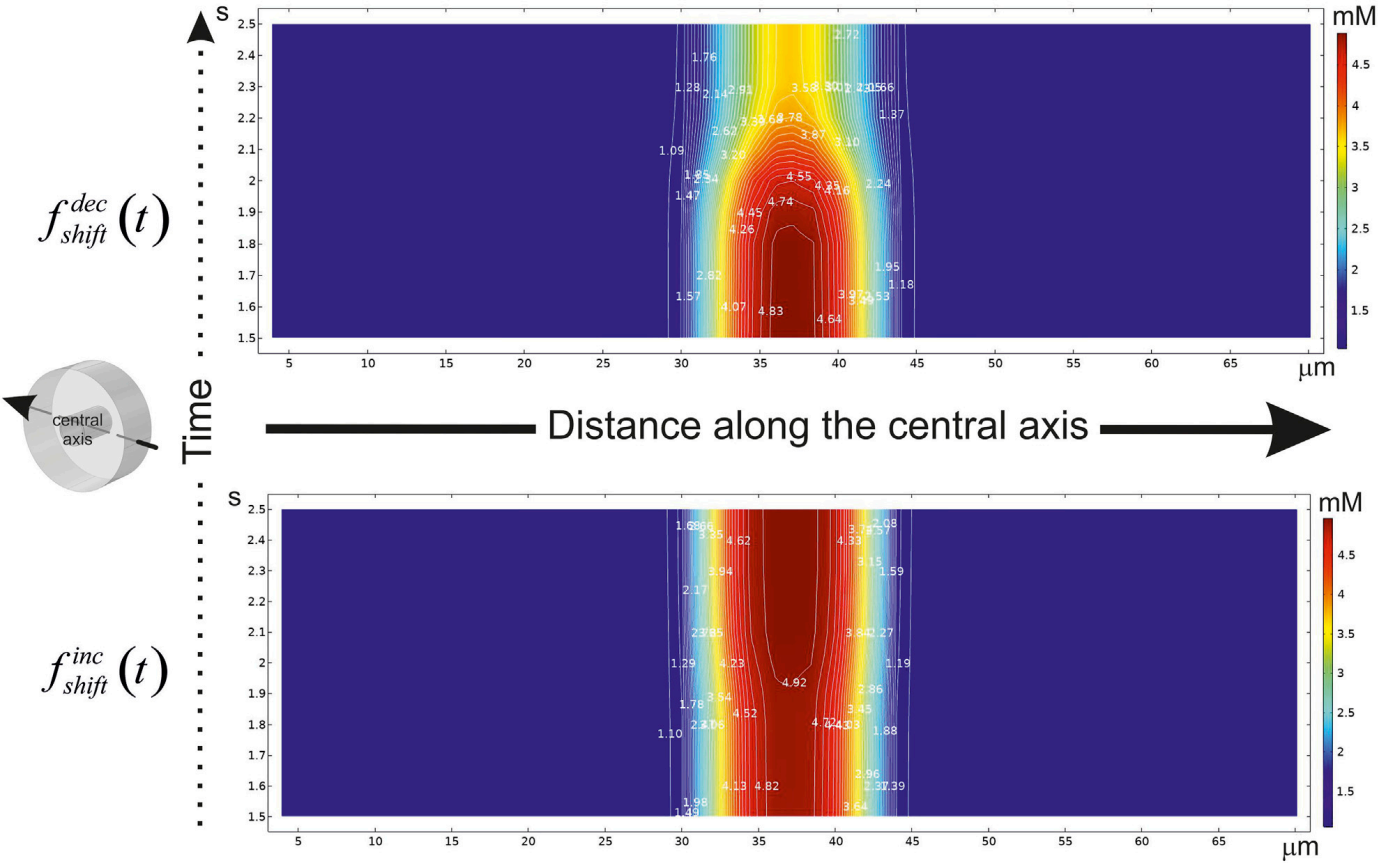
A diagram of the glucose concentration changes after CBF shift in time. The abscissa indicates the distance (μm) along the central axis penetrating the virtual phantom at mid-points of X and Y. The axis position is hinted in an isometric illustration in the left. The ordinate indicates the time scale (s). If the step-like function had been applied, CBF either to be decreased ( $f_{shift}^{dec}\left(t\right)$ , top) or to be increased ( $f_{shift}^{inc}\left(t\right)$ , bottom). White lines with the numbers draw the isolines of the same glucose concentration. The diagrams are represented for $\delta_{end-feet}$ = 85.6%.

The chosen parameters of the pressure in a considered blood vessel yield the appropriate modeled values of velocity magnitude $\langle \vec{u}\rangle$ (1.28 ± 0.06 mm/s) in compare to the experimental results (1.60 ± 0.70 mm/s (Hudetz et al., 1996); 2.03 ± 1.42 mm/s (Unekawa et al., 2010); 0.99 ± 0.17 mm/s (Lyons et al., 2016)); Simultaneously, the velocities calculated with governing Eq. 4 (4.1 ± 0.5 × 10^–7^ m/s ) are also in a good relation to the measured values of ISF in approximately 5 × 10^–7^ m/s (Zhan et al., 2017). It should be noticed that the velocity field has been formed as a gradient in both $\Omega_{I}$ and $\Omega_{II}$ . The introduced approach makes it possible to evaluate not only a mean value of $\vec{u}$ , but a distribution of this variable can be also represented for analysis. An application of $f_{shift}^{dec/inc}\left(t\right)$ to the pressure on the end transverse surfaces ${S^{\prime }}_{\partial \Omega_{I}}\;$ and ${S^{\prime \prime }}_{\partial \Omega_{I}}\;$ modifies a blood flow forming a decreased or an increased CBF. These changes cause an essential alteration of a spatial-time distribution of glucose concentration in a capillary lumen. The example of such a gradient is shown in Figure 5.

The essential variance is localized in a smoothing area near the point of shift. Indeed, the main influence on the gradient in $\Omega_{I}$ is accomplished by velocity field changes comparing to reaction-diffusion. It is remarkable that a blood flow velocity has a considerable impact on glucose gradients in a tissue part of the phantom, but the changes of this metabolite level in brain parenchyma crucially depend on the value of $\delta_{end-feet}$ . If the end-feet cover the basal lamina continuously with a small free diffusion area on $H_{\partial \Omega_{II}}^{inner}$ ( $\delta_{end-feet}$ is high) than the changes in glucose gradients in $\Omega_{II}$ seems to be simply predictable.

**FIGURE 6:**
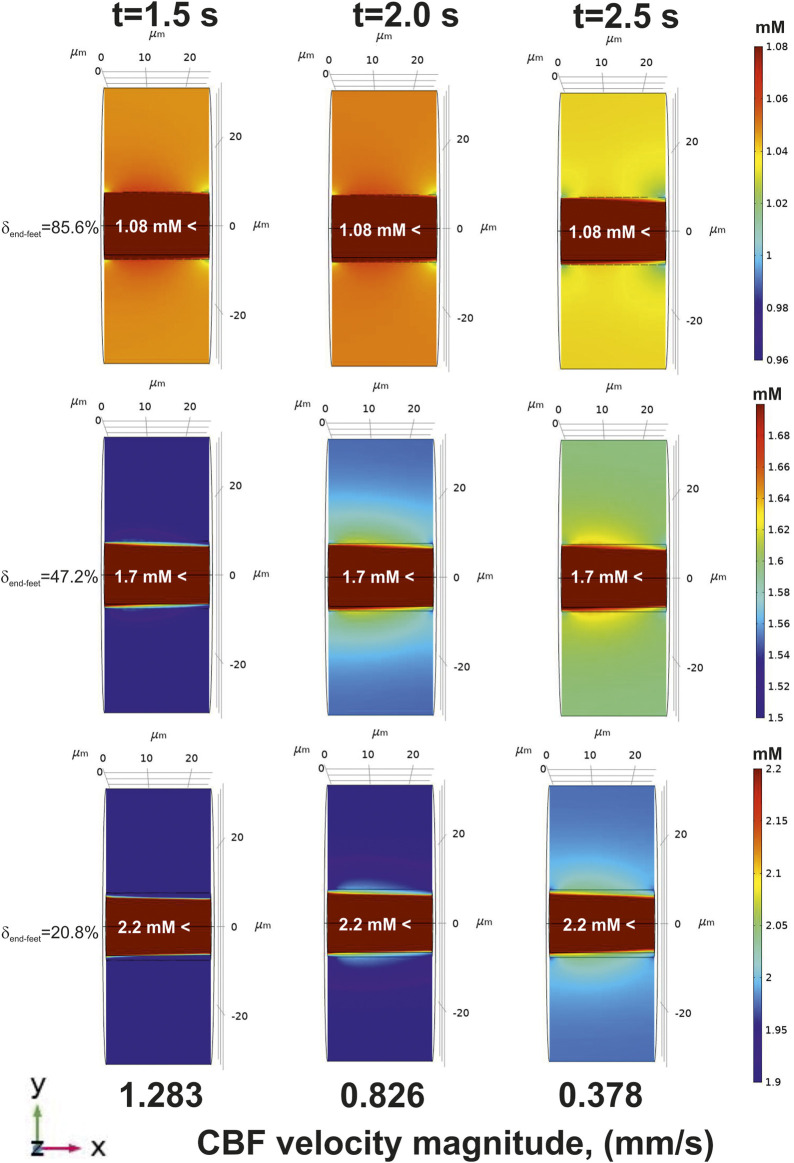
The glucose gradients near a blood capillary surface after CBF decrease (an application of $f_{shift}^{dec}\left(t\right)$ to the pressure on the end transverse surfaces ${S^{\prime }}_{\partial \Omega_{I}}\;$ and ${S^{\prime \prime }}_{\partial \Omega_{I}}\;$ ). The concentrations are represented in the central longitudinal cut plain XY at the three moments of time (indicated in the top). The corresponding CBF velocity magnitudes are specified in the bottom. For clear visualization the different glucose concentration scales are chosen in each case. The lumen of blood capillary ( $\Omega_{I}$ ) has a separate distribution of glucose formed by reaction-diffusion and convection. Due to a narrow range of scale, it is filled with a single color and a numerical indication. The glucose gradients represented for 85.6%, 47.2%, and 20.8% of $\delta_{end-feet}$ respectively.

Decrease of an incoming glucose causes a lowered level of this metabolite in the tissue part (Figure 6, top). However, if the area of a free diffusion from the basal lamina is high ( $\delta_{end-feet}$ is low), a slow-down velocity of CBF breeds an increased amount of glucose in $\Omega_{II}$ (Figure 6, medium and bottom).

**FIGURE 7:**
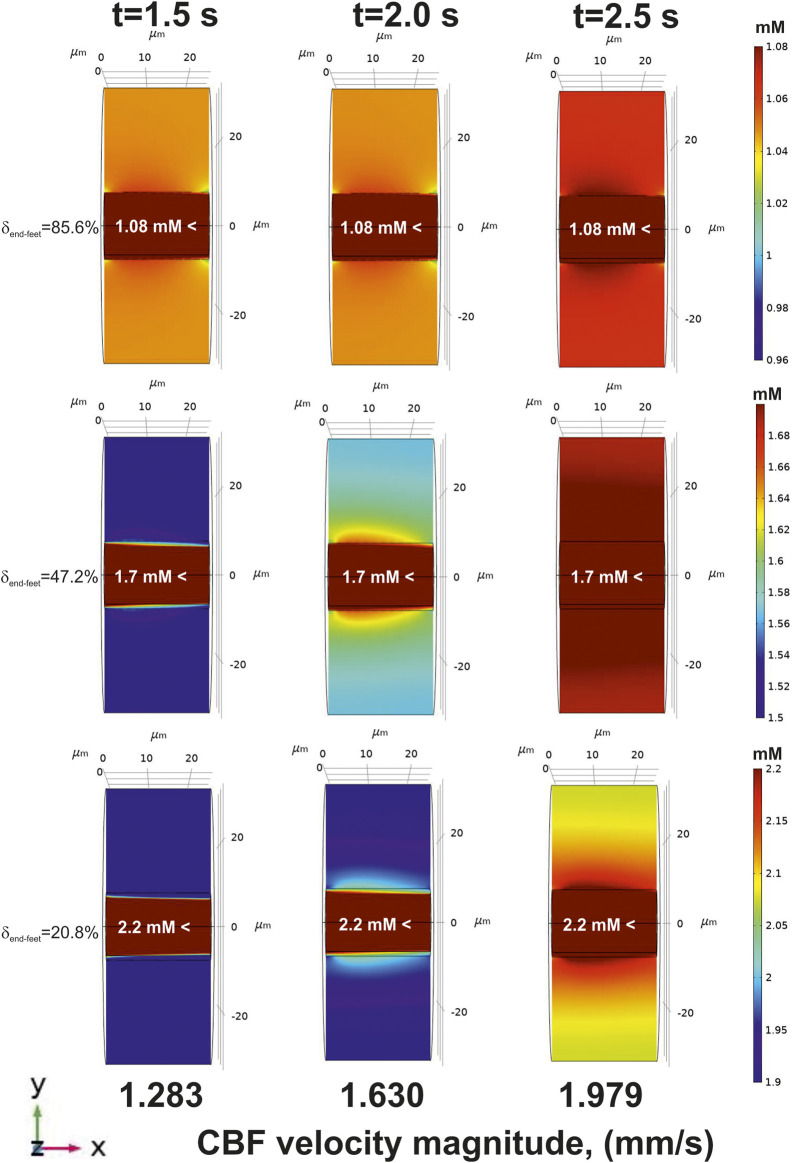
The glucose gradients near a blood capillary surface after CBF increase [application of $f_{shift}^{inc}\left(t\right)$ ]. All designations are the same as in Figure 5.

On the contrary, there is no such a difference between $\delta_{end-feet}$ in the case of $f_{shift}^{inc}\left(t\right)$ . An increased CBF always cause an enhanced level of glucose in $\Omega_{II}$ (Figure 7). The structure of $H_{\partial \Omega_{II}}^{inner}$ also influence the amplitude of a glucose gradient in $\Omega_{II}$ . An average value of glucose level in a nervous parenchyma part of the phantom is variated from 1.03 ± 0.02 mM ( $\delta_{end-feet}$ =85.6%) to 2.2 ± 0.3 mM ( $\delta_{end-feet}$ =20.8%) which is in a good relation to the experimentally measured value of 1.7 ± 0.9 mM (Choi et al., 2001).

## 5 Discussion

**FIGURE 8:**
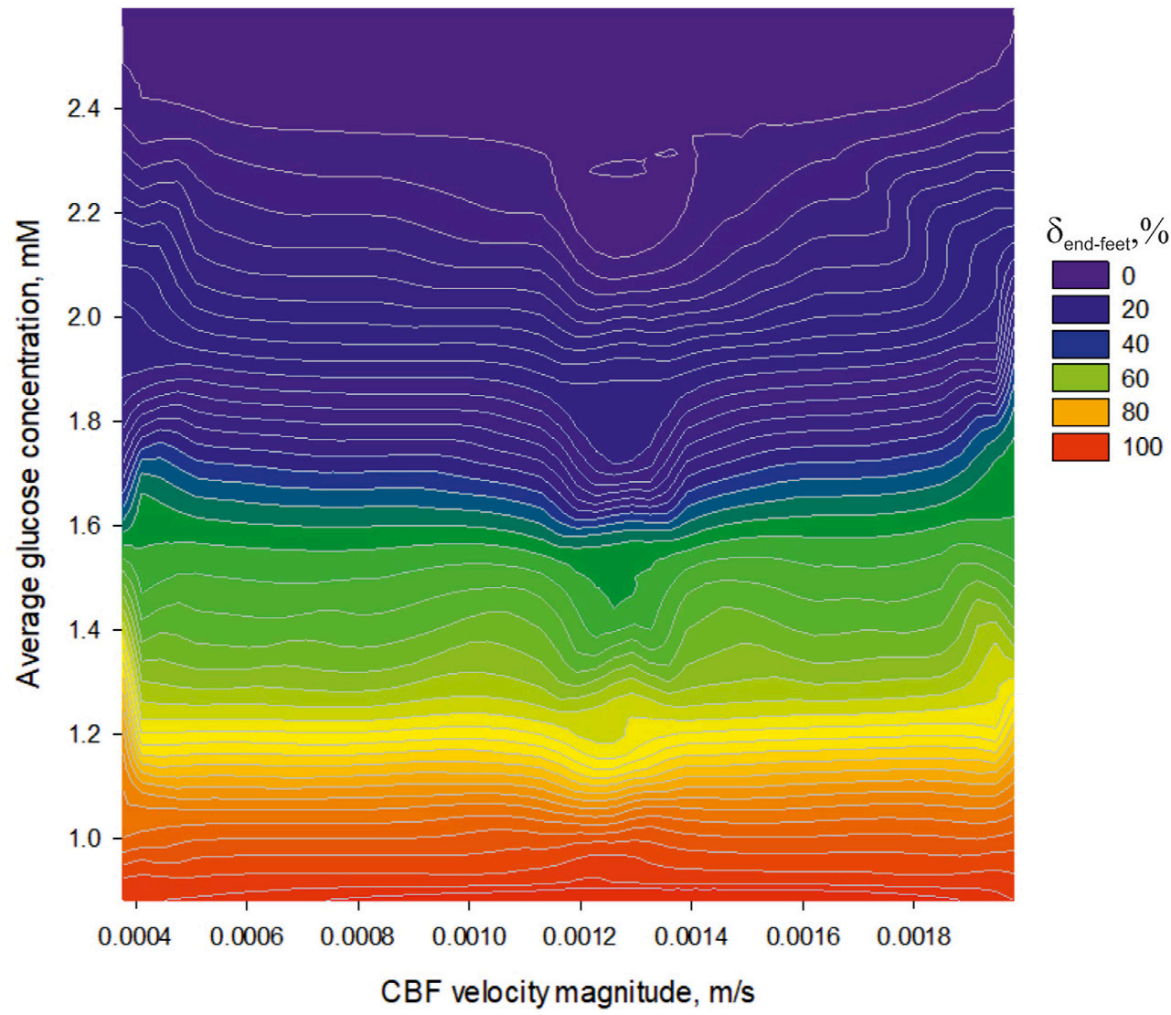
The dependence of an average glucose concentration in nervous parenchyma ( $\Omega_{II}$ ) near a capillary surface on CBF velocity magnitude ( $\Omega_{I}$ ) under different percent of the end-feet cover.

The results of the introduced multyphysical approach reveal an especial physiological feature of BBB. In a classic view BBB is created by a tight junction between endothelial cells that form the walls of the capillaries (Abbott et al., 2010). Nevertheless, astrocytes also sealed the area around a blood vessel by the end-feet network. Certainly, there is a complex structure of the clefts between the astrocytes where nutrients can penetrate fluently. According to the methodology of the present study it means in that area glucose incomes by a simple diffusion. The existence of a free diffusion area intermitting end-feet was experimentally observed (Hawkins et al., 2006). Moreover, the simulations predicted a very close correspondence between theory and data obtained in rats and humans (Gruetter et al., 1998; Choi et al., 2001) only under conditions when $\delta_{end-feet}$ <100% (Simpson et al., 2007). Indeed, the proportion of GLUTi/free diffusion areas can be a subject of discussion, however, an enhanced level of glucose due to CBF reduction is already observed under the condition of $\delta_{end-feet}\leq 85\%$ (Figure 8). Thus, the part of the third internal obstacle for a free diffusion can be relatively high to make the effect visible.

A physical background of this phenomenon has previously been explained (Nartsissov, 2021a). The increased CBF always causes an enhanced amplitude of the glucose gradient in $\Omega_{II}$ because the level of this metabolite will continuously raise up with an incoming flow according to Eq. 12. The extremum values of CBF velocity magnitude in Figure 8 can be explained by the optimum relation between GLUT density, $H_{\partial \Omega_{II}}^{outer}$ area and $c\left(\vec{r},t\right){|}_{\Omega_{I}}$ . Indeed, the relation depends on a blood vessel dimeter, but the general effect will be the same.

The introduced model has different physiological applications which help clinicians to investigate the aspects of neuropathology. First, the geometry of the phantom Ω can be easily reshaped. The structural principles of NVU organization will be the same for a long-type capillary and even for a vessel network. Furthermore, the diameter of a capilllary is also among the parameters to be modified. The small blood capillary vessels are usually ranged from 5 to 10 μm in diameter (Vendel et al., 2019; Xing et al., 2020). However, a distinction of caliber between arterioles, pre-capillaries and capillary is blur, and the range of a capillary network with the diameter up to 15 μm is also reported (Fleischer et al., 2020). During experimental studying of a cerebral capillaries functional reactivity, the vessels with diameter above 10 μm and below 30 μm are classified as the medium-size vessels (Stefanovic et al., 2008). Due to a normal human red blood cell has the shape of a biconcave disk with a diameter of approximately 8 μm and a thickness of approximately 2 μm (Secomb, 2017), a short length of a small capillary can get some especial properties resulting from a vessel wall friction. That is why in the present study the geometry of the considered system is designed to the upper range of a capillary diameter (R_0_, see Table 1). Due to the surrounding capillaries get pronounceable effects on glucose gradients near the external boundary of the virtual phantom ( $H_{\partial \Omega_{II}}^{outer}$ ), an extension of the phantom by inclusion of a vasculature network is preferable. It is remarkable that arterioles and capillaries can be included in the model all together. The modification of the general phantom structure should be made by an addition of a smooth muscle wall for arterioles which is represent as $\Omega_{SMW}$ placed between $\Omega_{E}$ and $\Omega_{BL}$ . The reconstruction of a vascular network can be fulfilled by either digital proceedings of experimental images or especially created blood vessels tree phantoms. The principles of digital arterial network creation has recently been discussed elsewhere (Kopylova et al., 2017). An application of the step-like functions $f_{shift}^{dec/inc}\left(t\right)$ to the pressure on the end transverse surfaces of the network can simulate a brain circulation disorder with further alteration of the glucose levels inside nervous parenchyma.

Moreover, the developed approach can be used for estimation of CBF alteration influence on several types of metabolites. For oxygen and nitric oxide, the model will be simplified by the omitting of the flux discontinuity on the internal borders, but the other principles remain the same. For the drugs, the structure of the flux dysconnectivity functions in Eq. 15 should be modified. Additionally, the third summand in the second member of Eqs 5, 6 can be simplified to a linear dependence. However, as in the case of oxygen, the main described algorithm will persist the same.

Another advantage of the model is to evaluate a wide set of physical parameters which are useful for medical purposes. The considered CBF velocities can be transformed into the standard unit of measurement for CBF which is milliliters of blood per 100 g of tissue per minute (Liu, 2015). The considered time area in $f_{shift}^{dec/inc}\left(t\right)$ connecting with short-term (>0.20 Hz) fluctuations in CBF velocity. They closely match those observed in arterial pressure and likely reflect mechanical/biophysical properties of the cerebrovascular bed in response to changes in arterial pressure (Zhang et al., 2000). However, Δt can be extended to several seconds, and this time range is associated with cerebral autoregulation which does not depend on changes in arterial pressure.

Nevertheless, impaired cerebral autoregulation leads to dependence of blood flow on blood pressure, which may affect blood supply to brain when peripheral blood pressure is reduced under physiological and pathological conditions (Hu et al., 2008). This phenomenon is also the subject of consideration in the introduced model.

Another aspect of the introduced approach is a detailed consideration of brain parenchyma—the area $\Omega_{II}$ . For general purpose it can be approximated by a porous media. This description is commonly used in the most spatial-time models (Syková and Nicholson, 2008). Nevertheless, there are several metabolites which have an essential difference in diffusion inside astrocyte/neurons area and ISF. A well-known example of such metabolites is glutamate (Nartsissov, 2022). This neuromediator has a strong regulated level in the inter-cell space and the most part of its concentration is stored into astrocytes (Selivanov et al., 2021). For modeling of spatial-time gradients of such neurotransmitters the explicit form of cell-to-cell position in parenchyma should be designed. It can be done using an algorithm based on 3D Voronoi diagram application (Nartsissov, 2021b). The obtained structure will have a drawn area for diffusion and convection. It causes spatial complication of $\Omega_{II}$ but more precise effects become accessible.

Thus, the introduced method is an adaptive tool for analysis of CBF changes influence on spatial-time gradients of glucose. It can be also used for evaluation of non-steady state spatial distribution of different chemical compounds. Certainly, the supposed model can be applied not only to modeling of brain parenchyma but also for estimation of the gradients near blood vessels in other tissues as well.

## Data Availability

The original contributions presented in the study are included in the article/Supplementary Material, further inquiries can be directed to the corresponding author.
